# Electrical Conductivity, Thermo-Mechanical Properties, and Cytotoxicity of Poly(3,4-Ethylenedioxythiophene):Poly(Styrene Sulfonate) (PEDOT:PSS)/Sulfonated Polyurethane Blends

**DOI:** 10.3390/ma17184602

**Published:** 2024-09-19

**Authors:** Gagan Kaur, Gavin E. Collis, Raju Adhikari, Pathiraja Gunatillake

**Affiliations:** CSIRO Manufacturing, Bayview Avenue, Clayton, VIC 3168, Australia; gavin.collis@csiro.au (G.E.C.); adhikari2428@gmail.com (R.A.)

**Keywords:** conductive composites, polymeric films, ionic, segmented, biocompatibility, synthesis, compatibility, neural implants

## Abstract

Electrically conductive polymeric materials have recently garnered significant interest from researchers due to their potential applications in the biomedical field, including medical implants, tissue engineering, flexible electronic devices, and biosensors. Poly(3,4-ethylenedioxythiophene):poly(styrene sulfonate) (PEDOT:PSS) is considered the most successful conducting polymer due to its higher electrical conductivity and chemical stability, but it suffers from limited solubility in common organic solvents, poor mechanical properties, and low biocompatibility. An area of tremendous interest is in combining PEDOT:PSS with another polymer to form a blend or composite material in order to access the beneficial properties of both materials. However, the hydrophilic nature of PEDOT:PSS makes it difficult to produce composites with non-polar polymers. In order to overcome these problems, we have specifically designed and synthesized two new sulfonated polyurethanes (PUS) with high sulfonic acid functionality. The two polyurethanes, one water-soluble (PUS1) and one water-insoluble (PUS2), were used to make blends with two commercially available PEDOT:PSS formulations (Clevios^TM^ FET and PH1000). Solvent cast films on glass substrates were made from water-soluble PEDOT:PSS/PUS1 blends while free-standing films of PEDOT:PSS/PUS2 blends were fabricated by compression-moulding. Ethylene glycol was used as conductivity enhancer, which showed an increase in the conductivity by several orders of magnitude in most of the compositions investigated. The highest conductivity of 438 S cm^−1^ was achieved for the blend with 80 wt% of PEDOT:PSS (PH1000) in PUS1.

## 1. Introduction

Electrically conductive polymeric (ECP) materials have drawn significant attention for their promising applications in biomedical implants, stretchable electronics, biosensors, intelligent robotics, body-conformable wearable devices, and material science applications [1,2,3]. A variety of doped conducting polymers, based on polyaniline, polypyrrole, and poly(3,4-ethylenedioxythiophene) (PEDOT), have been developed for these diverse applications [1,2,4,5,6]. Poly(3,4-ethylenedioxythiophene):poly(styrene sulfonate) (PEDOT:PSS) is widely regarded as one of most successful ECPs, thanks to its high electrical conductivity and chemical stability, which make it highly suitable for use in organic electronics, biomedicine and biotechnology [7,8]. PEDOT:PSS is commercially available in the form of an aqueous solid dispersion which can be processed into thin films by solution and solid-state methods. PEDOT:PSS is synthesized by aqueous oxidative polymerization of the 3,4-ethylenedioxythiophene (EDOT) monomer in the presence of the template polymer poly (styrene sulfonate) (PSS). Polymerization with excess oxidant sodium peroxodisulfate yields the PEDOT:PSS complex in a cationic doped conductive form. PSS anion is a commercially available, water-soluble polymer which serves two functions in the PEDOT:PSS complex; it acts as a charge-balancing counterion to the PEDOT cation and a dispersant/surfactant for the PEDOT segments in water [9]. PEDOT:PSS has attracted significant interest in the field of bioelectronics and neural electrodes due to its low toxicity with several cell types [10,11].

Although PEDOT:PSS has enormous potential as an organic conductive material, there are some limitations; poor solvent compatibility, poor processability, poor mechanical properties, low biofunctionality, and biocompatibility have restricted its widespread commercial use [1,12]. Furthermore, the direct use of only PEDOT:PSS seems to have long-term issues such as PEDOT chain degradation and the possible release of acidic PSS degradation by-products. Two main approaches have been reported in the literature to overcome or minimise these issues. One approach is to change the composition of PEDOT:PSS by either modifying the β-position of the EDOT monomer or replacing PSS with biopolymers, while the second strategy is to use PEDOT:PSS together with another polymer in the form of a blend or composite in order to access the useful properties of both materials [13,14].

Several biopolymers, such as DNA, sulfated cellulose, dextran sulfonate, hyaluronic acid, heparin, chondroitin sulfate, and guar gum, have been explored in the polymerization of EDOT in order to enhance the biocompatibility and reduce the cytotoxicity of the resulting electroactive PEDOT [14,15,16,17,18]. The electro-polymerization using various biopolymers as counteranions is a common approach producing several different water-based PEDOT:biopolymer dispersions. While many of these trials have achieved polymerization of EDOT, the conductivity of the resulting doped PEDOT:biopolymer films is often very low. In fact, most of these PEDOT:biopolymer films have conductivity values of <10 S cm^−1^ [14,19].

A more practical and easier way of overcoming some of the limitations of PEDOT:PSS has been to make hybrid systems by blending with a non-conducting polymer to improve mechanical performance and biocompatibility [20,21,22]. In such systems, the ECP’s role is to impart conductivity to the blend, while the non-conducting polymer renders the material mechanical properties. Polyurethanes (PUs) are of particular interest to make ECP blends because of their biocompatibility, biostability, processability, and good mechanical properties [23,24]. Several reports can be found in literature on conducting polymer/PU blends, with most studies primarily focusing on their mechanical and electrical properties [22,25,26]. Notably, the majority of PUs used in these studies are water-dispersible, which poses a challenge as films or coatings made from water-dispersible PU may degrade or disintegrate in aqueous environments, thereby limiting their applicability in such conditions. For an in situ grown ECP PEDOT:PU blend, a high conductivity of 100 S cm^−1^ was demonstrated, even under an elongation of >100% [27]. In general, the electrical conductivity of the ECP is compromised to achieve improved mechanical properties by blending with a mechanically strong non-conducting polymer. Overall, the composite/blend approach has had some success, but the low loading levels of the conductive material have prohibited access to the desirable hybrid properties of these blends. Thus, elaborate material design is necessary to fulfil the material property specifications required for specific applications.

The main aim of our study was to make conductive blends of PEDOT:PSS with a sulfonated polyurethane (PUS) derivative for potential use in biomedical applications. A key area of interest in developing these conductive polymeric materials is their potential use in neural implants, such as conducting polymer electrodes designed for neural applications to stimulate cells to treat neurological conditions like epilepsy. This study aimed to produce polymeric blends with enhanced processability and improved mechanical properties without significantly compromising their conductivity. Although blends of PEDOT:PSS with polyurethanes have been explored previously, blends of PEDOT:PSS with sulfonated polyurethanes have not been studied before. It was proposed that the ionic nature of the PUS systems would enhance compatibility with PSS to yield a more homogeneous blend without significantly compromising overall electrical conductivity. For this purpose, two sulfonated polyurethane systems, one water-soluble (PUS1) and one water-insoluble (PUS2), were synthesized and evaluated. The water-insoluble PUS2 was prepared for its potential use in aqueous environments without disintegrating. We also investigated the effectiveness of ethylene glycol, a well-known conductivity enhancer, on the overall conductivity of these PEDOT:PSS/PUS blends. Furthermore, this study not only examined the electrical and mechanical properties but also investigated the biocompatibility of the synthesized sulfonated polyurethanes.

## 2. Experimental

### 2.1. Materials

The PEDOT:PSS (Clevios^TM^ FET and Clevios^TM^ PH1000) was used as received. The poly (ethylene glycol) (PEG) (M_n_ = 400, Sigma Aldrich, Castle Hill, Australia) was dried at 105 °C under a vacuum (10 mbar) to remove any volatile impurities. The isophorone diisocyanate (IPDI) (98%, Sigma Aldrich, Castle Hill, Australia), hexamethylenediisocyanate (HDI) (≥98%, Sigma Aldrich), dibutyltin dilaurate (95%, Sigma Aldrich, Castle Hill, Australia), and zinc acetate dihydrate (GR grade, Acros Organics, Geel, Belgium) were used as received. The dimethyl 5-sulfoisophthalate sodium salt (98%, Sigma Aldrich, Castle Hill, Australia) was dried overnight in an oven at 100 °C prior to use.

Benzidine-2,2′ disulfonic acid (BDSA) (Tokyo Chemical Industry Co., Ltd., Tokyo, Japan) was of technical grade which was purified and converted to its sodium salt as described elsewhere [28]. Briefly, the as-received BDSA was washed with hot water to remove any water-soluble contaminants and filtered. This procedure was repeated until the filtrate was clear. The filter cake was titrated with an aqueous sodium hydroxide solution. The endpoint of this titration is easily detected since the acid form has a very low solubility in water while the sodium ion form is highly soluble in water. The resulting solution was filtered to remove any particulate matter and concentrated by solvent evaporation. The sodium salt of BDSA (BDSA-Na) was precipitated in acetone. The precipitates were filtered, washed with cold acetone, and dried.

### 2.2. Synthesis of Sulfonated Polyurethanes

#### 2.2.1. Synthesis of Sulfonated Polyol (SP)

**Scheme 1:**
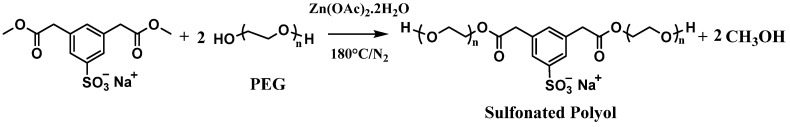
Synthesis of sulfonated polyol.

Sulfonated polyol was synthesized according to the method outlined in Scheme 1 described elsewhere [29,30]. Dimethyl 5-sulfoisophthalate sodium salt (18.67 g 0.063 mol) and PEG (50.00 g, 0.125 mol) were placed in a round bottom flask, and zinc acetate dihydrate was subsequently added as a catalyst (0.05 wt%). The mixture was stirred at 180 °C for 16 h under nitrogen to produce sulfonated polyol. The molecular weight of the polyol was determined using the hydroxyl number method and ^1^H NMR.

#### 2.2.2. Synthesis of Sulfonated Polyurethanes (PUS1)

The synthesis of water-soluble polyurethane (PUS1) was carried out using two-step polymerization in DMAc solvent. The degassed sulfonated polyol (5.018 g, 4.54 mmol) was dissolved in 25 mL of DMAc, and IPDI (2.116 g, 9.52 mmol) was added to the polyol solution while stirring. After the IPDI addition was complete, 2 drops of dibutyltin dilaurate were added to the reaction mixture and the reaction was continued for 15 h at 70 °C under nitrogen while stirring. After 15 h, the temperature of the reaction was reduced to 40 °C and BDSA-Na (1.761 g, 4.54 mmol) was added along with 22–25 mL of DMAc. The reaction was further continued under nitrogen for 3 days at 40 °C while stirring. The viscous product was transferred to a Teflon mould, and the solvent was evaporated under vacuum at 50 °C to obtain the polymer. The molecular weight was determined using GPC.

#### 2.2.3. Synthesis of Sulfonated Polyurethane (PUS2)

The polyurethane synthesis of PUS2 was carried out using two-step polymerisation in DMAc as a solvent. The degassed sulfonated polyol (6.028 g, 6.02 mmol) was dissolved in 4 mL of DMAc, and HDI (1.966 g, 11.69 mmol) was added to the polyol solution while stirring. After the HDI addition was complete, 2 drops of dibutyltin dilaurate were added to the reaction mixture and the reaction was continued for 1.5 h at 80 °C under nitrogen while stirring. After 1.5 h, the temperature of the reaction was reduced to 40 °C and BDSA-Na (2.117 g, 5.45 mmol) was added along with 22–25 mL of DMAc. The reaction was further continued under nitrogen for 40 h at 40 °C while stirring. The viscous product was transferred to a Teflon mould, and the solvent was evaporated under vacuum at 50 °C to obtain the polymer. The molecular weight was determined using GPC.

### 2.3. Preparation of PEDOT:PSS/PUS Films

#### 2.3.1. Preparation of PEDOT:PSS/PUS1 Solvent Cast Films on Glass

**Figure 1:**
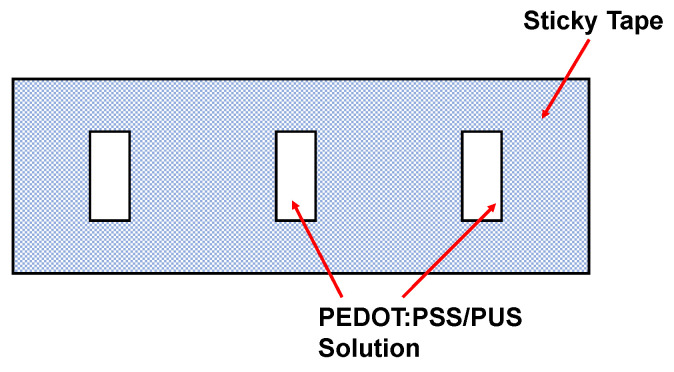
Preparation of solvent cast films on a glass slide.

A stock solution (5 wt%) of water-soluble polyurethane PUS1 was prepared. The PEDOT:PSS/PUS1 blends were prepared by mixing the appropriate amounts of the stock PUS1 solution with 2 mL of either of two PEDOT:PSS formulations (PH1000 and FET). The blends were sonicated for 4–5 min and further stirred (using a magnetic spin-bar) for at least 15 min to obtain uniformly mixed polymer blends. Various polymer blends were prepared with varied loadings of PEDOT:PSS in the range of 20–80 wt%. To prepare solvent cast films, recesses (2 × 1 cm) were created on a glass microscope slide using an adhesive tape as shown in Figure 1. In total, 100 µL of each polymer blend solution was dispensed into the recess. The slide was rotated manually to evenly coat the surface. Excess solution was tipped off the slide. For ethylene glycol treatment, ethylene glycol (6 vol%) was added to polymer blend solutions which were then processed in the same way as described above to prepare solvent cast films. The coatings were dried at 105 °C for 10 min and further at 115 °C for 5 min in an oven. The sticky tape was removed, and the thickness of the resultant coatings measured using a Dektak 150 Stylus Profilometer (Veeco Instruments Inc., Tucson, AZ, USA). The coating thickness was determined from the height difference as obtained from line scans over coated and non-coated surfaces. The average of six measurements was taken at different locations of each specimen; the readings varied in the range 2–8 µm for all samples, indicating the thickness was not uniform across the film.

#### 2.3.2. Preparation of Compression-Moulded Films

A stock solution of PUS2 (3 wt%) was prepared in DMAc. The PUS2 stock solution was mixed with one of the two PEDOT:PSS dispersions (FET and PH-1000) in different proportions to obtain polymer blend mixtures with varied loadings of PEDOT:PSS. Ethylene glycol was added to the mixtures (10 vol%) which were then sonicated for 4–5 min before being further stirred for at least 15 min to facilitate mixing. After stirring, the polymer blends were transferred to PTFE moulds (100 × 100 × 2 mm) and were dried under vacuum at 70 °C overnight. The dried solids were collected and films (of approximately 150 ± 50 µm thickness) were obtained by melt-pressing. The melt-pressing was performed at 125 °C under a nominal load of ~8 tonnes over 0.04 m^2^ for 2 min, followed by cooling to ambient temperature via circulation of cold water through the platens.

### 2.4. Methods

#### 2.4.1. Polyol Characterisation

The molecular weight of sulfonated polyol was analysed by determining the hydroxyl number using the reflux phthalation method in accordance with the ASTM D4274 standard [31]. Proton Nuclear Magnetic Resonance (^1^H NMR) spectra were recorded at 400 MHz with a Bruker Av400H spectrometer (Bruker Corp., Billerica, MA, USA). The NMR spectra refer to solutions in deuterated chloroform (CDCl_3_), where the solvent signal for residual protons was used as an internal standard.

#### 2.4.2. Polymer Characterisation

Molecular weights of polyurethanes were obtained using gel permeation chromatography (GPC), performed on a Shimadzu system equipped with a CMB-20A controller system, SIL-20A HT autosampler, LC-20AT tandem pump system, DGU-20A degasser unit, CTO-20AC column oven, RDI-10A refractive index detector, and 4 × Waters Styragel columns (HT2, HT3, HT4, and HT5, each 300 mm × 7.8 mm^2^, providing an effective molar mass range of 100 to 4 × 10^6^). The eluent was *N*,*N*-Dimethylacetamide (DMAc) containing 0.05 mol L^−1^ lithium bromide (LiBr), with a flow rate of 1 mL min^−1^ at 80 °C. A series of polystyrene standards with molecular weights ranging from 575 to 3,242,000 g mol^−1^, were used for calibrating GPC columns, and the number average molecular weights (M_n_) are reported as polystyrene equivalents.

Fourier transform infrared (FTIR) spectra were collected on a Perkin Elmer Spectrum 2000 FTIR instrument (PerkinElmer Inc., Waltham, MA, USA) in attenuated total reflectance (ATR) mode using diamond as the background reference.

#### 2.4.3. Measurement of Conductivity

Conductivity measurements were performed on PEDOT:PSS/PUS samples of approximately 2 × 1 cm in size. Conductive silver paint was used for facilitating the electrical contact. After the silver paint electrode deposition, DC resistance measurements were carried out in two-probe configuration using a Keithley 2400 Source Meter (Keithley Instruments Inc., Cleveland, OH, USA) by driving current and measuring the voltage. Surface electrical conductivity was calculated using the method described below.

The resistivity *ρ*, in Ω cm, was calculated, using the following equation:

*ρ* = (R × L × d)/a = (V × L × d)/(I × a), in Ω cm

where R = electrical resistance in Ω, L = electrode length (cm), d = film thickness (cm), a = electrode separation (cm), V = voltage in Volts, and I = current in Amp.

The conductivity *σ* was calculated using the following relationship:

*σ* = 1/*ρ*, in S cm^−1^

The conductivity values are reported as the average of three replicates.

#### 2.4.4. Thermogravimetric Analysis (TGA)

The TGA analyses were conducted on a Mettler Toledo TGA/SDTA 851 thermogravimetric analyser, and data were collected and processed using STARe software (version 15.00). Samples of the materials (1–10 mg) were precisely weighed into open alumina crucibles (70 μL). The analyses were conducted over a temperature range of 30–600 °C with a heating rate of 10 °C/min in a nitrogen atmosphere (nitrogen flow rate: 30 mL/min).

#### 2.4.5. Differential Scanning Calorimetry (DSC)

The thermal transitions of the materials were analysed using differential scanning calorimetry (DSC). Prior to measurements, all samples were dried under vacuum at 70 °C for 48 h to eliminate moisture. Analyses were performed on a Mettler Toledo DSC 821 with polymer samples (~5 mg) using 40 µL aluminum pans. The calibration was performed using the indium/zinc total method. The measurements were conducted at temperatures from −50 to 280 °C at a heating rate of 10 °C/min under nitrogen (flow rate = 30 mL/min). The STARe software was used to determine glass transition temperatures (Tg) from temperature versus heat flow plots.

#### 2.4.6. Scanning Electron Microscopy (SEM)

SEM images were taken using a ZEISS MERLIN Ultra High Resolution field emission SEM (FE-SEM) instrument (ZEISS Microscopy, Jena, Germany) operated at an accelerating voltage of 5–20 kV. For surface morphology, the samples were coated with approximately 200 Å of iridium before analysis. The cross-sectional analysis was performed on cryogenically fractured samples coated with ~250 Å thick carbon.

#### 2.4.7. Tensile Testing

Tensile testing of PEDOT:PSS/PUS2 films (of approximately 150 ± 50 µm thickness) was performed using ASTM D882 [32], a standard test method for evaluating the tensile properties of thin plastic sheeting. The samples were prepared as dumbbell-shaped specimens, measuring 3 cm in length and 1 cm in width, with a narrower section of 1.2 cm by 0.4 cm. The specimens were conditioned under ambient conditions for 4 weeks before testing. The tests were performed on an Instron 5565 testing machine using a 1 kN load cell and a crosshead speed of 10 mm/min. The results presented are the averages of five replicates. The same procedure was applied to neat sulfonated polyurethane (PUS) specimens, which served as the control.

#### 2.4.8. Assessment of the In Vitro Cytotoxicity (Indirect Cytotoxicity Test)

The indirect cytotoxicity testing of polyurethane films was performed using a protocol based on ISO 10993-5 ‘Biological evaluation of medical devices—Part 5: Tests for in vitro cytotoxicity’ [33,34]. The samples were placed in the culture medium at 37 °C for 72 h and samples of the medium (hereafter referred to as extracts) were collected from each of the samples. The extracts were prepared in a dilution series and placed onto L929 cells that had been pre-seeded into wells of a 96-well culture plate. The viability of cells in quadruplicate wells of each polymer treatment was quantified after 24 h using a 3-(4,5-dimethylthiazol-2-yl)-2,5-diphenyltetrazolium bromide (MTT) assay. An outcome from the MTT assay which resulted in a reduction in cell viability of greater than 30% was deemed to be cytotoxic. The cell culture controls for this cell-based assay included cells seeded into uncoated wells in serum-free medium (SFM) and 5% phosphate-buffered saline (PBS) in serum-free medium (as positive cell controls) and 5% dimethylsulfoxide (DMSO) in serum-free medium (as a control cell-killer). Representative bright field images of each dilution tested were taken at 20 h, prior to the addition of the MTT, to check cell morphology.

## 3. Results and Discussion

### 3.1. Synthesis of Sulfonated Polyol and Polyurethanes

**Figure 2:**
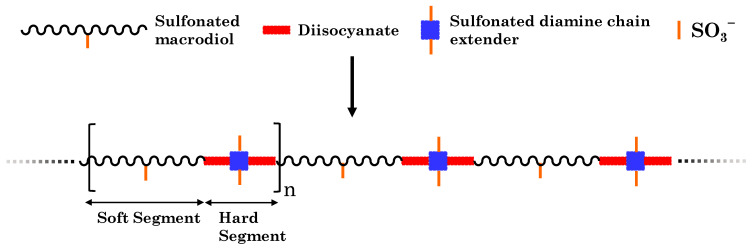
Composition/structure of sulfonated polyurethane.

Two sulfonated polyurethanes, water-soluble (PUS1) and water-insoluble (PUS2), were synthesized by the introduction of sulfonate groups in soft segments as well as hard segments (Figure 2). For the hard segment, a sulfonated chain extender, benzidine-2, 2′disulfonic acid sodium salt (BDSA-Na), and a diisocyanate (IPDI or HDI) was used. For the soft segment, a sulfonated macrodiol was synthesized by the transesterification of dimethyl 5-sulfoisophthalate sodium salt with polyethylene glycol (PEG). The molecular weight (M_n_) of PEG used to synthesize sulfonated macrodiol was 400.

The molecular weight of the sulfonated macrodiol was determined using the hydroxyl number method (ASTM D4274 standard). The molecular weight was found to be 1105 which was in agreement with literature-reported values [29,30]. This was also confirmed by ^1^H NMR spectroscopy by integrating the peak area corresponding to the aromatic protons of dimethyl 5-sulfoisophthalate sodium salt (Ar-H, δ = 8.6–8.8) against protons corresponding to PEG (-CH_2_-CH_2_-O-, δ = 3.5–3.8) (Supplementary Information, Figure S1). The M_n_ from ^1^H NMR was calculated to be 1101 which corresponded well with the M_n_ obtained from the hydroxyl number method.

**Scheme 2:**
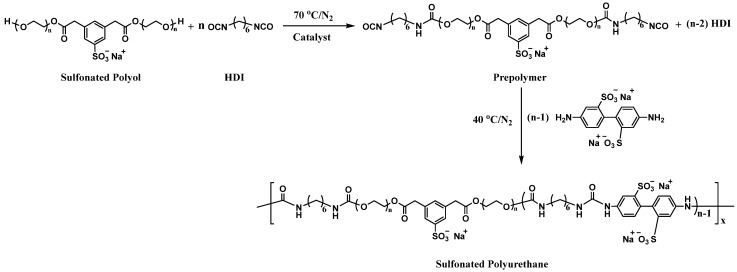
Synthetic route to a sulfonated polyurethane PUS2.

PUS1 was synthesized using isophorone diisocyanate (IPDI), while hexamethylenediisocyanate (HDI) was used to synthesize PUS2. The polyurethanes were synthesized by the two-step polymerization method in DMAc as a solvent. In the first step, a prepolymer was formed from sulfonated polyol and a diisocyanate (IPDI or HDI). The sulfonated polyurethanes were obtained by addition of the sulfonated chain extender (BDSA-Na) to the prepolymer in the second step. Scheme 2 presents the synthetic route to PUS2. The hard segment weight % was 43.6% in PUS1 and 40.4% in PUS2. The wt% of sulfonate groups (SO_3_^−^), as calculated from the feed ratio of the starting materials, was 12.24% in PUS1 and 12.94% in PUS2. The products were characterized using GPC and FTIR. The molecular weights of polymers were determined using GPC in DMAc as a mobile phase. The molecular weight of PUS1 was 27,970 (PDI = 1.43) and that of PUS2 was 57,220 (PDI = 1.79).

The FTIR transmittance plots of the synthesized PUS showed a band around 1540 cm^−1^ corresponding to urethane N-H bending and C-N stretching which confirmed the formation of urethane linkage in both polyurethanes (Supplementary Information, Figure S2). Furthermore, the absence of a peak at 2250–2270 cm^−1^ corresponding to free isocyanate confirmed the complete consumption of starting materials in both polyurethanes.

### 3.2. PEDOT:PSS/PUS Films

PUS1 was used for the preparation of solution-cast conductive coatings on glass slides, while PUS2 was used in the preparation of free-standing conductive films. Four blend series were prepared with their compositions presented in Table 1.

#### 3.2.1. Conductivity of PEDOT:PSS/PUS1 Films on Glass Substrates

Two series of PEDOT:PSS/PUS1 blends containing varied loadings of PEDOT:PSS were prepared using two commercially obtained PEDOT:PSS formulations, Clevios^TM^ FET and Clevios^TM^ PH1000. These blend series are henceforth denoted as PH1000/PUS1 and FET/PUS1 (Table 1). The loading of PEDOT:PSS in the blends was varied from 20 to 100 wt%.

**Figure 3:**
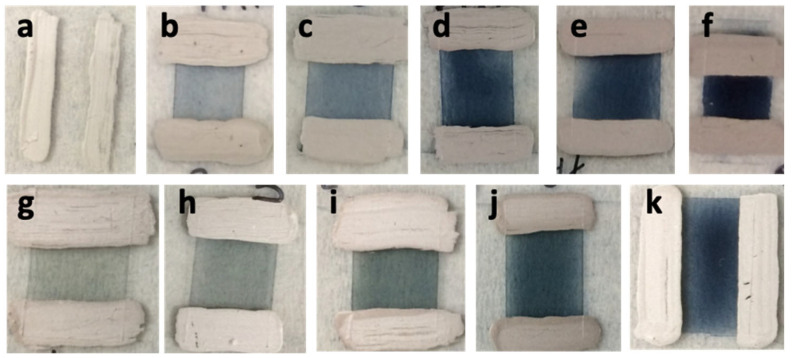
PEDOT:PSS/PUS1 (EG-treated) solvent cast films on glass slides with Ag electrodes (Ag paint) deposited on them for conductivity measurements. (**a**) Neat PUS1, (**b**) 20% PH1000/PUS1, (**c**) 40% PH1000/PUS1, (**d**) 60% PH1000/PUS1, (**e**) 80% PH1000/PUS1, (**f**) neat PH1000, (**g**) 20% FET/PUS1, (**h**) 40% FET/PUS1 (**i**) 60% FET/PUS1, (**j**) 80% FET/PUS1, and (**k**) neat FET.

The two PEDOT:PSS formulations Clevios^TM^ FET and Clevios^TM^ PH1000 were chosen because of the difference in their conductivity. The supplier reported that the conductivity of the Clevios^TM^ PH1000 film is 850 S cm^−1^ (after treatment with 5% dimethylsulfoxide (DMSO)), whereas that of the untreated Clevios^TM^ FET film is 200 S cm^−1^. It is worth mentioning here that the conductivity of PEDOT:PSS films can be significantly enhanced by secondary doping which involves addition of a high-boiling polar organic solvent, such as DMSO, ethylene glycol (EG), nitromethanol, and diethylene glycol, in a small amount as reported in the literature [35,36]. Although the mechanism of conductivity enhancement by secondary doping is not fully understood, it is widely believed that secondary doping causes phase segregation between PEDOT and PSS [35,37,38]. The phase segregation leads to the conformational change of the PEDOT chains from a coil structure to an extended-coil or linear structure resulting in enhanced conductivity, presumably extending the conducting pathway. Furthermore, the conductivity of as-prepared (untreated) Clevios^TM^ PH1000 films has been reported to be ~1 S cm^−1^, whereas the conductivity of these films after treatment with EG is reported to be in the range of 600–700 S cm^−1^ [37,39]. For these reasons, EG (6 vol%) was added to PEDOT:PSS/PUS1 aqueous blends prior to film casting in order to enhance the conductivity. Both series, PH1000/PUS1 and FET/PUS1, were prepared with and without EG treatment to compare the effect of addition of EG on conductivity. The dried PEDOT:PSS/PUS1 films obtained were 2–8 µm thick with a uniform and smooth appearance (Figure 3). Furthermore, the films containing <80 wt% of PEDOT:PSS were semitransparent.

**Figure 4:**
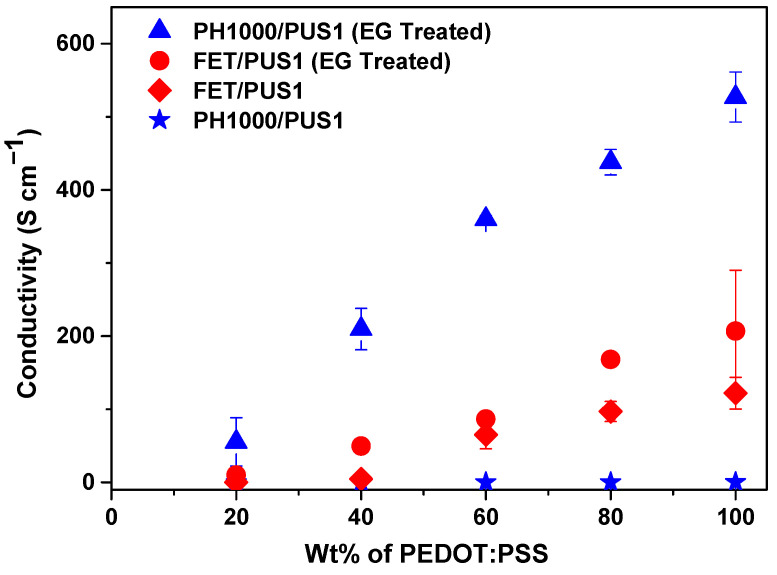
Electrical conductivity of PEDOT:PSS/PUS1 solvent cast films on glass substrates.

The electrical conductivity of PEDOT:PSS/PUS1 films was measured in a two-probe configuration. The conductivity results of PH1000/PUS1 and FET/PUS1 films, with and without EG treatment, are presented in Figure 4. As expected, the conductivity of both PEDOT:PSS/PUS1 films increased with the increasing loading of PEDOT:PSS. The conductivity of neat PH1000 without EG treatment was ~0.35 S cm^−1^ which is comparable to literature reports [37,39]. The conductivity of PH1000/PUS1 films changed from 0.005 S cm^−1^ for 20 wt% loading of PEDOT:PSS to 0.046 S cm^−1^ for 80 wt% loading of PEDOT:PSS. For the FET/PUS1 films, the conductivity of neat FET without EG treatment was 122 S cm^−1^ and the conductivity of FET/PUS1 films increased from 0.029 S cm^−1^ for 20 wt% loading PEDOT:PSS to 97 S cm^−1^ for 80 wt% loading of PEDOT:PSS.

The conductivity of PEDOT:PSS/PUS1 blends was significantly enhanced with the addition of EG for both of the PEDOT:PSS formulations (Table 2). For the PH1000/PUS1 films, the highest conductivity achieved was 438 S cm^−1^ for EG-treated blends containing 80 wt% loading of PEDOT:PSS, while the blends with only 20 wt% of PEDOT:PSS exhibited a reasonably good conductivity of 55 S cm^−1^. For the FET/PUS1 films, the highest conductivity achieved was 168 S cm^−1^ for EG-treated blends containing 80 wt% loading of PEDOT:PSS, while the blends with 20 wt% loading of PEDOT:PSS showed a conductivity of 10 S cm^−1^. Furthermore, the enhancement in conductivity was significantly higher (up to two orders of magnitude) for FET/PUS1 films containing ≥60 wt% of PUS1 than those with lower content of PUS1 (see Table 2). For the EG-treated PH1000/PUS1 films, the conductivity of blends with ≤20 wt% of PUS1 increased by three orders of magnitude while the conductivity of blends containing ≥40 wt% of PUS1 increased by four orders of magnitude.

The above results clearly show that the enhancement in conductivity is significantly higher for blends with a lower PUS1 content. As mentioned above, the addition of EG presumably enhances the conductivity due to the structural ordering of the PEDOT and PSS phases, but these results also indicate that the sulfonated polyurethane is likely to be acting as a compatibilizer and further facilitates greater uniform integration of all components favouring better conductive pathways within the polymer matrix. Furthermore, the conductivity of 40 wt% PH1000/PUS1 films was 210 ± 28 S cm^−1^, which was higher than the previously reported values for blends of PEDOT:PSS with conventional polyurethanes [22,40]. Choong and co-workers fabricated stretchable electrodes from polymer blends of PH1000 and a polyurethane treated with conductivity enhancers [22]. These blends exhibited a conductivity of ~168 S cm^−1^ for samples containing 40 wt% of PEDOT:PSS [22]. In another similar work [40], blends of a non-ionic waterborne polyurethane with PH1000 were prepared which possessed a conductivity of ~185 S cm^−1^ for the same amount (40 wt%) of loading. In comparison to these studies, we have achieved a higher conductivity for comparable PU loading—perhaps indicating that further enhancement in conductivity may be achieved by optimizing the formulation.

#### 3.2.2. Conductivity of Free-Standing PEDOT:PSS/PUS2 Films

**Figure 5:**
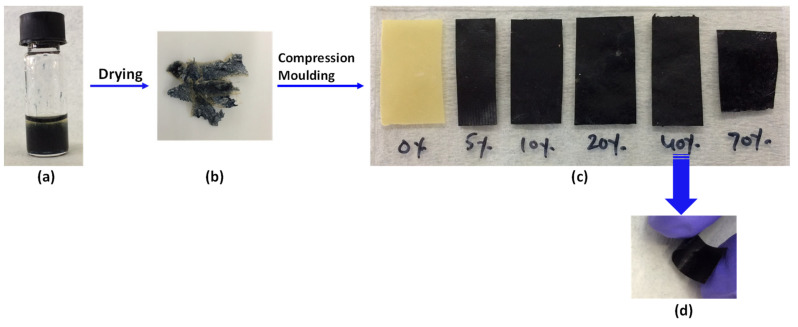
(**a**) 5% PH1000/PUS2 blend mixture in DMAc. (**b**) 5% PH1000/PUS2 dried solid. (**c**) PH1000/PUS2 (EG-treated) compression-moulded films with different loadings of PH1000. (**d**) 40% PH1000/PUS2 flexible film.

Based on the above results obtained for EG-treated PEDOT:PSS/PUS1 films, blends of PEDOT:PSS and a water-insoluble sulfonated polyurethane (PUS2) were prepared with EG treatment and were used to prepare free-standing films. Two series of PEDOT:PSS/PUS2 blends were prepared using two formulations (PH1000 and FET) and the loading of PEDOT:PSS in each series was varied from 5 to 100 wt%. The free-standing PEDOT:PSS/PUS2 films (Figure 5) were prepared by the compression-moulding method, and their conductivity was also measured using a two-probe configuration as described above.

**Figure 6:**
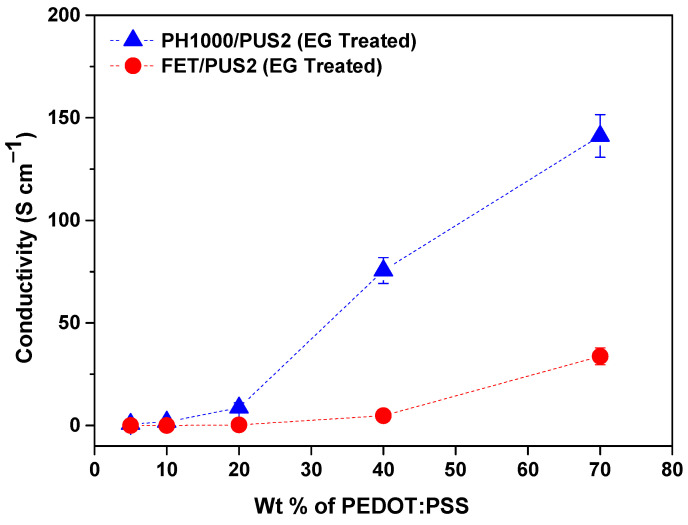
Electrical conductivity of PEDOT:PSS/PUS2 free-standing films.

The conductivity data of PEDOT:PSS/PUS2 films (of approximately 150 ± 50 µm thickness) are presented in Figure 6. For the PH1000/PUS2 films, the highest conductivity achieved was 141 S cm^−1^ for films containing 70 wt% loading of PEDOT:PSS, while the films with only 5, 20, and 40 wt% of PEDOT:PSS exhibited a conductivity of 0.072, 8.7, and 76 S cm^−1^, respectively. These conductivity values were significantly less than those of solvent cast films having same composition. For FET/PUS2 films, the conductivity was <1 S cm^−1^ for films with ≤20 wt% loading of FET which increased to 4.8 S cm^−1^ for sample with 40 wt%. A maximum conductivity of 34 S cm^−1^ was achieved for FET/PUS2 films with 70 wt% loading of PEDOT:PSS.

The conductivity of the PEDOT:PSS/PUS2 films was significantly less than that of the PEDOT:PSS/PUS1 films which may be a result of the poor mixing of the components due to the incompatibility between PEDOT:PSS and non-water-soluble PUS2. Although the conductivity of films obtained from compression-moulding method was less when compared to that of solvent cast films on glass substrates, the observed results are very encouraging. The use of PUS2 as a host polymer and the compression-moulding method provided free-standing water-insoluble films with impressive conductivity even with ≥20 wt% loading of PEDOT:PSS. The free-standing PEDOT:PSS/PUS2 films (EG-treated) were also characterized for their morphology, mechanical properties, thermal properties, and cytotoxicity, the results of which are discussed in the following sections.

### 3.3. Morphology of PEDOT:PSS/PUS2 Films

**Figure 7:**
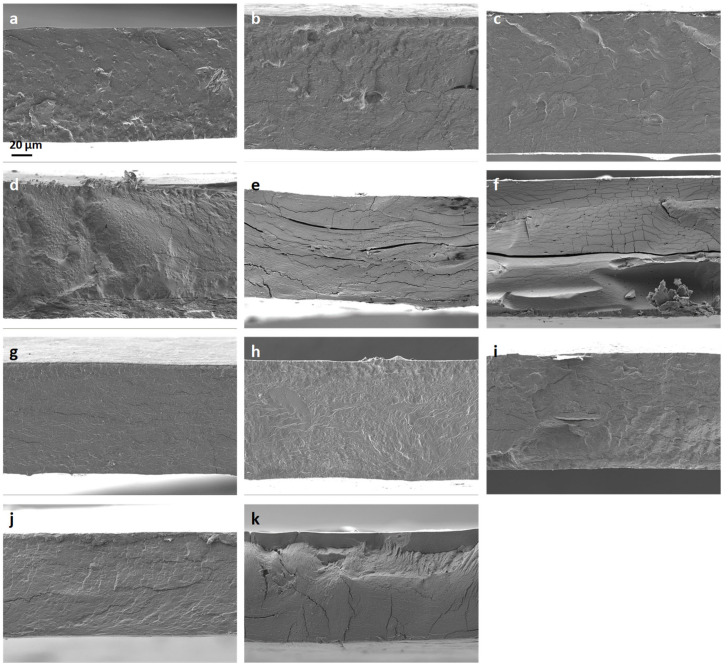
Cross-sectional FE-SEM images of compression-moulded PEDOT:PSS/PUS2 (EG-treated) films at magnification of 500: (**a**) neat PUS2, and with PH1000 loadings of (**b**) 5%, (**c**) 10%, (**d**) 20%, (**e**) 40%, and (**f**) 70%, and with FET loadings of (**g**) 5%, (**h**) 10%, (**i**) 20%, (**j**) 40%, and (**k**) 70%.

**Figure 8:**
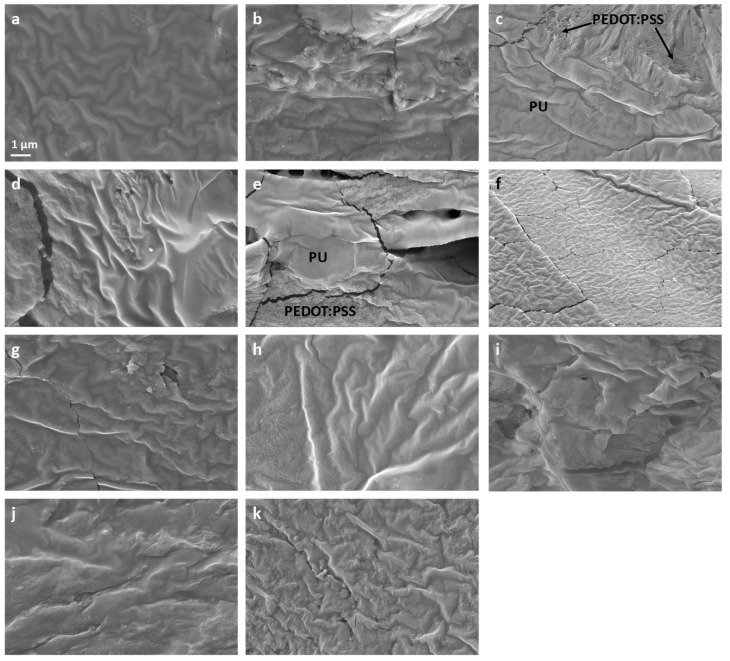
Cross-sectional FE-SEM images of compression-moulded PEDOT:PSS/PUS2 (EG-treated) films at magnification of 10,000: (**a**) neat PUS2, and with PH1000 loadings of (**b**) 5%, (**c**) 10%, (**d**) 20%, (**e**) 40%, and (**f**) 70%, and with FET loadings of (**g**) 5%, (**h**) 10%, (**i**) 20%, (**j**) 40%, and (**k**) 70%.

The cross-sectional FE-SEM images of cryogenically fractured PEDOT:PSS/PUS2 films are shown in Figure 7 and Figure 8. Figure 7 shows images taken at a magnification of 500. The SEM images showed a uniform morphology confirming that PEDOT:PSS was evenly incorporated into the PUS matrix. However, cracks were seen in the blends which were more severe at higher loadings of PEDOT:PSS. The cracks were also visible in neat PUS2, suggesting that the cryogenic fracturing treatment during sample preparation could have induced cracking in the microstructure. However, the cracks were larger and more severe in PH1000/PUS2 films with loadings of >20 wt% of PEDOT:PSS than the cracks seen in FET/PUS2 films. This suggests that the composition of PEDOT:PSS itself (i.e., composition of PH1000 and FET formulations) could also be responsible for the presence of cracks. In comparison to PH1000/PUS2, the morphology of FET/PUS2 films was uniform with minimal cracking in blends with PEDOT:PSS loading of up to 40 wt%. 

The SEM images were also taken at a higher magnification of 10,000 (Figure 8) to further examine the mixing of components in the blends. The PUS2 regions were marked with a smooth texture, while the PEDOT:PSS regions were of a rougher/flaky appearance (Figure 8a,c,e). The SEM images at higher magnification also confirmed the uniform incorporation of PEDOT:PSS in the polyurethane matrix, with minor aggregation of PEDOT:PSS observed for PH1000/PUS2 films (Figure 8c,e). The morphology of the FET/PUS2 films was uniform throughout the matrix confirming an even mixing of components of the blend. There were no signs of aggregation in the FET/PUS2 films.

### 3.4. Mechanical Properties of PEDOT:PSS/PUS2 Films

The mechanical properties of PEDOT:PSS/PUS2 films were characterized by tensile testing and are summarized in Table 3. The tensile strength of blends increased with increasing loading of PEDOT:PSS, while their elasticity decreased. The tensile strength of neat PUS2 was 2.7 (±0.30) MPa and the elongation at break was 252 (±41)%. For FET/PUS2 films, the tensile strength remains unchanged upon addition of up to 10 wt% of FET but increased to 4 MPa with 20 wt% loading of FET and to 6.4 MPa for 70 wt% loading of FET. The elongation (at break) was 174% for films containing 5 wt% of FET and 90% for films with 10 wt% loading of FET which was 31% and 64%, respectively, less than the property of the parent material. Furthermore, the films containing 20 wt% of FET were also reasonably elastic with an elongation of 56%; however, upon incorporation of 40 wt% of FET, the elasticity dropped significantly resulting in elongation of 19%. Furthermore, FET/PUS2 films with 70 wt% loading were comparatively rigid with an elongation of only 7%. For the PH1000/PUS2 films, the addition of PH1000 to PUS2 resulted in a higher increase in its tensile strength compared to that of FET/PUS2 films. The tensile strength of 5% PH1000/PUS2 increased by 178% (4.8 MPa), while the tensile strength of 40% PH1000/PUS2 increased by 268% and that of 70% PH1000/PUS2 increased by more than five-fold (increased by 564%). On the other hand, the elasticity of PH1000/PUS2 films was significantly low with the increased loading of PH1000. The 5% PH1000/PUS2 had an elongation of 82% (67% loss when compared to neat PUS2) which fell under 20% for ≥20 wt% loading of PH1000. Overall, the PEDOT:PSS/PUS2 blends showed improved mechanical strength but with a significant loss in elasticity of PUS2.

### 3.5. Thermal Analyses of PEDOT:PSS/PUS2 Blends

**Figure 9:**
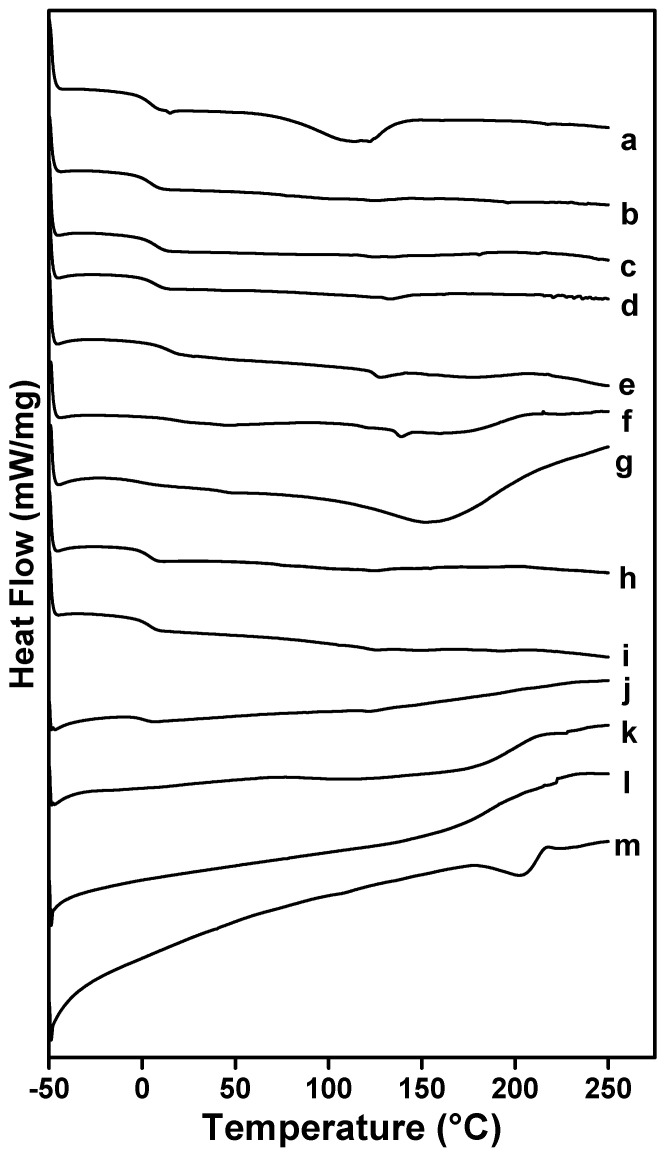
DSC plots of PEDOT:PSS/PUS2 blends. (a) Neat PUS2, (b) 5% FET/PUS2, (c) 10% FET/PUS2, (d) 20% FET/PUS2, (e) 40% FET/PUS2, (f) 70% FET/PUS2, (g) neat FET, (h) 5% PH1000/PUS2, (i) 10% PH1000/PUS2, (j) 20% PH1000/PUS2, (k) 40% PH1000/PUS2, (l) 70% PH1000/PUS2, and (m) neat PH1000.

The thermal stability of the PEDOT:PSS/PUS2 blends was examined by DSC and TGA. The DSC plots of neat PU and PEDOT:PSS/PUS2 blends with different loading of PEDOT:PSS are presented in Figure 9. For neat PUS2, the glass transition temperature (*T*_g_) corresponding to the soft segment was observed at 2.5 °C and a broad endothermic peak due to the hard segment melting was observed at ~113 °C (between 65 and 148 °C). The broad melting peak at ~113 °C also indicated the weak ordering of hard segment and, therefore, shows that PUS2 is mainly amorphous. The *T*_g_ appeared at the same temperature as that for the neat PUS2 for FET/PUS2 blends with up to 20 wt% loading. However, the *T*_g_ increased to ~12 °C for 40% FET/PUS2 and there was no clear soft segment *T*_g_ seen for 70% FET/PUS2. Similar observations were made for PH1000/PUS2 blends. Overall, the blends incorporated with up to 20 wt% of PEDOT:PSS appear to have no significant effect on the morphology of neat PUS. But the DSC evidence suggests some level of interaction between PUS2 chains and PEDOT:PSS particles for blends with higher loadings (≥40 wt%), leading to a change in the thermal transitions. The increased interaction may be the reason for the observed increase in the tensile strength of the compositions with higher loadings of PEDOT:PSS. 

**Figure 10:**
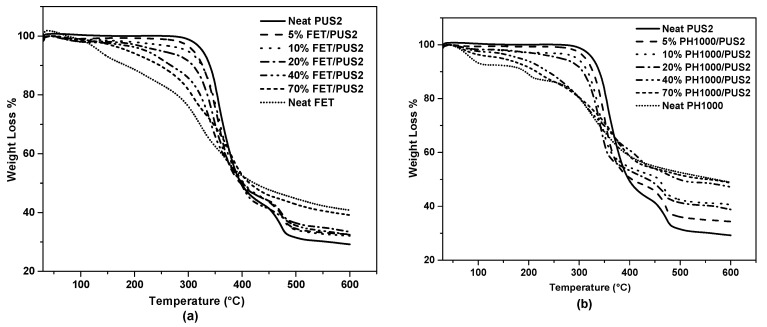
(**a**) TGA plots of FET/PUS2 blends with varied loading of FET. (**b**) TGA plots of PH1000/PUS2 blends with varied loading of PH1000.

Figure 10 presents the TGA plots of PEDOT:PSS/PUS2 blends containing varied amounts of PEDOT:PSS for both blends (PH1000/PUS2 and FET/PUS2). The neat PUS2 is thermally stable up to ~300 °C, while both the PEDOT:PSS formulations lose ~20–24% of their original mass at 300 °C. The TGA results of the blends showed that those materials containing up to 10 wt% loading of FET and PH1000 retained the thermal stability of the parent PUS2 material up to 275 °C. The blends with 20 wt% loading of PEDOT:PSS were thermally stable with <5% weight loss at temperatures of up to ~200 °C. The thermal stability of blends was further compromised with the increased loading of PEDOT:PSS (Table 4), however, all the blends were thermally more stable than both the neat PH1000 and FET formulations. The TGA results clearly show that the addition of PUS2 to PEDOT:PSS improves its thermal stability.

### 3.6. Cytotoxicity Testing of Sulfonated Polyurethanes (PUS1 and PUS2)

**Figure 11:**
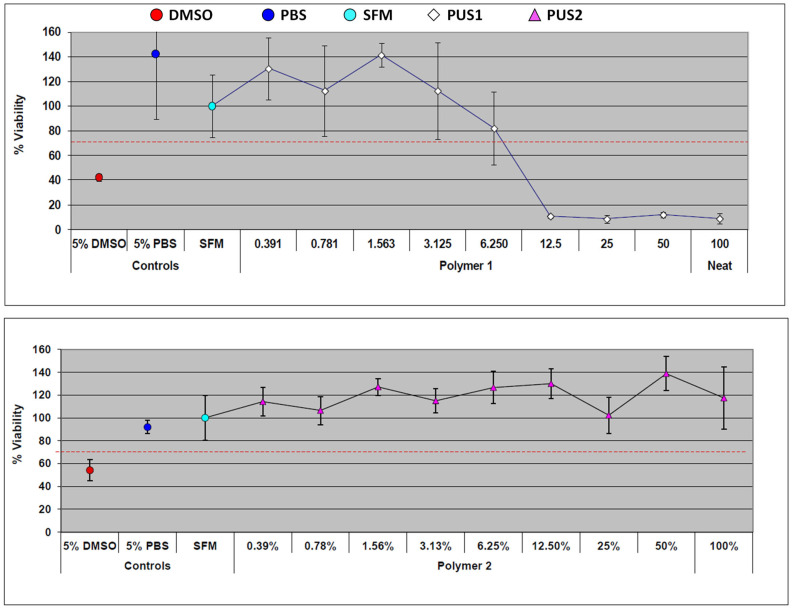
Viability of cells in serial dilutions of extracts of PUS1 (denoted Polymer 1 in plot) and PUS2 (denoted as Polymer 2 in plot) films (cell viability of 70% is marked by red dashed line and anything less than this is regarded to be cytotoxic). Cell culture controls included cells seeded into uncoated wells in SFM and 5% PBS in SFM as positive cell controls and 5% DMSO in SFM as a control cell-killer.

The outcome of the cytotoxicity assay conducted on extracts of the water-insoluble PUS2 showed that cell viability levels were at an acceptable level (above the 70% viability level) and equivalent to or better than the positive control serum-free medium (SFM) at every dilution of the PUS2 extract tested in the assay over the 24 h exposure period (Figure 11). Consistent with the cell viability outcomes, cell morphology was normal at all dilutions of the control PUS2 extract with cells looking similar to those in the SFM-positive control wells (Supplementary Information, Figure S3). Overall, the data showed that there was no evidence of cytotoxicity in the extract of the PUS2 polymer at the concentrations tested on live cells over the 24 h period. These results confirmed that no toxic leachable components were released from the PUS2 films during the 24 h extraction period.

This outcome contrasted that seen with the PUS1 polymer which was water-soluble and had completely dissolved in SFM during the 24 h extraction period. Cytotoxicity data showed that the extract of the control PUS1 polymer was cytotoxic to cells at dilutions of 12.5% (1:8) or stronger. The extract showed acceptable cell viability once dilutions were 6.25% (1:16) or weaker (Figure 11).

## 4. Conclusions

The results of this study supported the strategy of incorporating an ionic polyurethane to enhance the compatibility with PEDOT:PSS, producing processable uniform blends with conductivity directly dependent on the PEDOT:PSS loading. Furthermore, the results also supported that the use of the conductivity enhancer EG increases the conductivity by several orders of magnitude higher in most of the compositions investigated. The highest conductivity of 438 S cm^−1^ was achieved for the blend with 80 wt% of PEDOT:PSS (PH1000) in PUS1.

The use of water-insoluble PUS2 provided films with reasonable conductivity with ≥20 wt% loading of PEDOT:PSS. The ultimate tensile strength of the PUS2 blends was significantly higher compared to the neat PUS2, and the blend with 70 wt% loading of PH1000 showed the highest ultimate tensile strength at 15 MPa and a conductivity of 141 S cm^−1^. Furthermore, the material did not release any cytotoxic leachables as shown by the cytotoxicity assessment. The SEM results confirmed that films produced from FET compositions were more uniform compared to those prepared from PH1000. While the addition of an ionic polyurethane may not significantly enhance the electrical conductivity, the improvement in processability of the PEDOT-PSS is considerable—paving the way to broaden the application range where good mechanical strength and cell compatibility are required.

In summary, a number of PEDOT:PSS/PUS blends have been evaluated in this study that provide a combination of good tensile strength and electric conductivity with no evidence of cytotoxicity—all properties essential in a new material directed at biomedical applications such as coatings for neural implants. This study provides encouraging results to conduct further work to examine the following areas in detail: (a) the influence of the choice and loading of the conductivity enhancer (EG) on these chemical, physical, electronic, mechanical, and biological properties; (b) modification of water-soluble PUS1 to introduce post-fabrication cross links to reduce leachable components and improve biocompatibility; (c) strategies to increase the type and density of ionic groups to reduce or eliminate PSS in these compositions.

## Figures and Tables

**Table 1:** Composition of PEDOT:PSS/PUS blends.

Name of Blend Series	Type of PUS Used	PEDOT:PSS Formulation Used	PEDOT:PSS Loading (wt%) Range	Method of Preparation of Films
PH1000/PUS1	Water-Soluble PUS1	CleviosTM PH1000	20–100	Solvent cast
FET/PUS1	Water-Soluble PUS1	CleviosTM FET	20–100	Solvent cast
PH1000/PUS2	Water-Insoluble PUS2	CleviosTM PH1000	5–70	Compression-moulding
FET/PUS2	Water-Insoluble PUS2	CleviosTM FET	5–70	Compression-moulding

**Table 2:** Electrical conductivity of PEDOT:PSS/PUS1 solvent cast films.

Loading of PEDOT:PSS (wt%)	Conductivity without EG (S cm^−1^)	Conductivity with EG (S cm^−1^)	Change in Conductivity (Number-Folds Increase)
Neat PH1000	0.351 ± 0.077	530 ± 33	1511
80% PH1000	0.046 ± 0.009	438 ± 17	9520
60% PH1000	0.011 ± 0.001	360 ± 4.9	32,826
40% PH1000	0.008 ± 0.001	210 ± 28	25,265
20% PH1000	0.005 ± 0.001	55 ± 33	11,710
Neat FET	122 ± 22	207 ± 83	1.7
80% FET	97 ± 14	168 ± 2.3	1.7
60% FET	65 ± 19	87 ± 10	1.3
40% FET	4.7 ± 5.4	50 ± 8.6	10.6
20% FET	0.029 ± 0.01	10 ± 0.80	346

**Table 3:** Mechanical properties of compression-moulded PEDOT:PSS/PUS2 films.

Loading of PEDOT:PSS	Ultimate Tensile Strength, MPa (SD) *	Elongation at Break (%) (SD) *	Reduction in Elongation (versus PUS2)	Conductivity S cm^−1^
Neat PUS2	2.7 (0.3)	252 (41)	-	-
5% FET	2.6 (0.3)	174 (29)	31%	0.001
10% FET	2.5 (0.2)	90 (11)	64%	0.011
20% FET	4.0 (0.5)	56 (11)	78%	0.30
40% FET	5.7 (0.4)	19 (3.9)	92%	4.8
70% FET	6.4 (1.9)	7 (1.2)	97%	34
5% PH1000	4.8 (0.4)	82 (14)	67%	0.72
10% PH1000	7.2 (0.3)	26 (4.5)	90%	1.8
20% PH1000	5.4 (0.7)	10 (2.9)	96%	8.7
40% PH1000	7.2 (1.2)	5 (1.3)	98%	76
70% PH1000	15.2 (1.8)	6 (1.4)	98%	141

^*^ SD = Standard deviation.

**Table 4:** TGA data of PEDOT:PSS/PUS2 blends.

Blend Description	Weight Loss%		
	0–275 °C	275–450 °C	450–600 °C
Neat PUS2	0.1	58.5	12.1
5% FET/PUS2	1.5	54.9	11.1
10% FET/PUS2	3.7	52.5	11.7
20% FET/PUS2	6.2	49.8	10.6
40% FET/PUS2	10.8	47.9	8.8
70% FET/PUS2	13.5	40.7	6.7
Neat FET	19.4	32.8	6.9
5% PH1000/PUS2	1.2	53.2	11.2
10% PH1000/PUS2	3.6	45.4	10.5
20% PH1000/PUS2	5.6	46.0	9.5
40% PH1000/PUS2	15.1	31.0	6.7
70% PH1000/PUS2	16.2	29.8	5.3
Neat PH1000	15.6	29.6	5.9

## Data Availability

The original contributions presented in the study are included in the article/Supplementary Materials, further inquiries can be directed to the corresponding author.

## Supplementary Materials

The following supporting information can be downloaded at: https://www.mdpi.com/article/10.3390/ma17184602/s1, Figure S1: ^1^H NMR of sulfonated polyol. Figure S2: FTIR transmittance spectra of sulfonated polyurethanes. Figure S3: Representative images showing cell morphology after exposure to a diluting series of the PUS2 extract.
