# Supplementary material for: Electrical Conductivity, Thermo-Mechanical Properties, and Cytotoxicity of Poly(3,4-Ethylenedioxythiophene):Poly(Styrene Sulfonate) (PEDOT:PSS)/Sulfonated Polyurethane Blends
Source: Materials (Basel). 2024 Sep 19;17(18):4602. doi: 10.3390/ma17184602 (PMC11433249; doi:10.3390/ma17184602)
Supplement: Supplementary file 1 [file materials-17-04602-s001.zip › materials-3189515-supplementary.pdf]

Electrical conductivity, thermo-mechanical properties and cytotoxicity of Poly(3,4-ethylenedioxythiophene):poly(styrene sulfonate) (PEDOT:PSS)/sulfonated polyurethane blends

Gagan Kaur*, Gavin Collis, Raju Adhikari, Pathiraja Gunatillake*

CSIRO Manufacturing, Bayview Avenue, Clayton, VIC 3168, Australia

*Corresponding authors email: Gagan.Kaur@monash.edu, pgun5768@bigpond.net.au

SUPPLEMENTARY INFORMATION

NMR Data

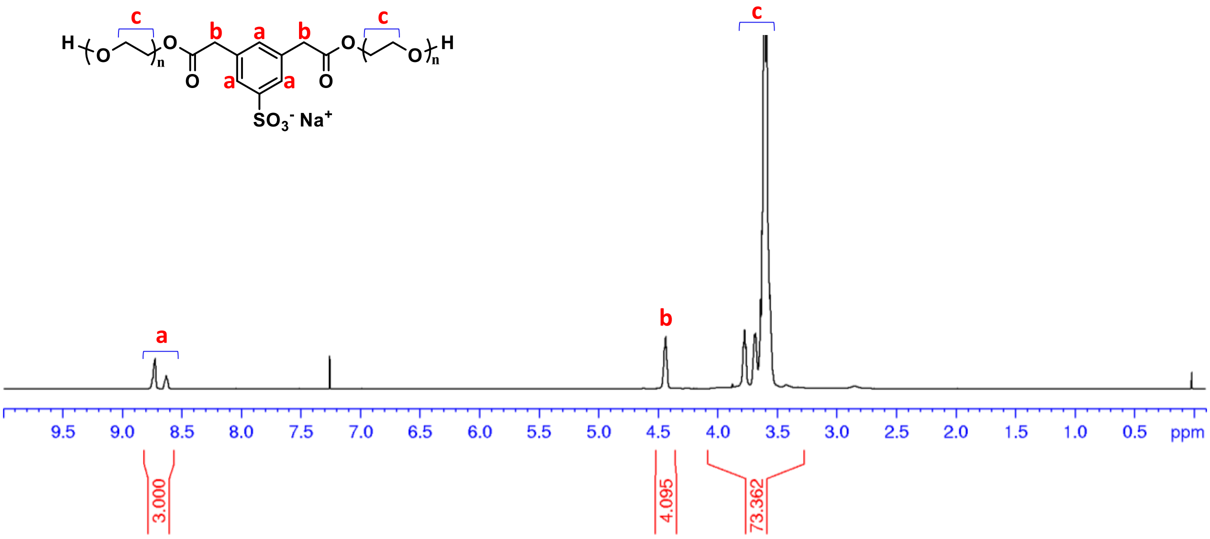

Figure S1: ^{1}H NMR of sulfonated polyol.

FTIR Plots

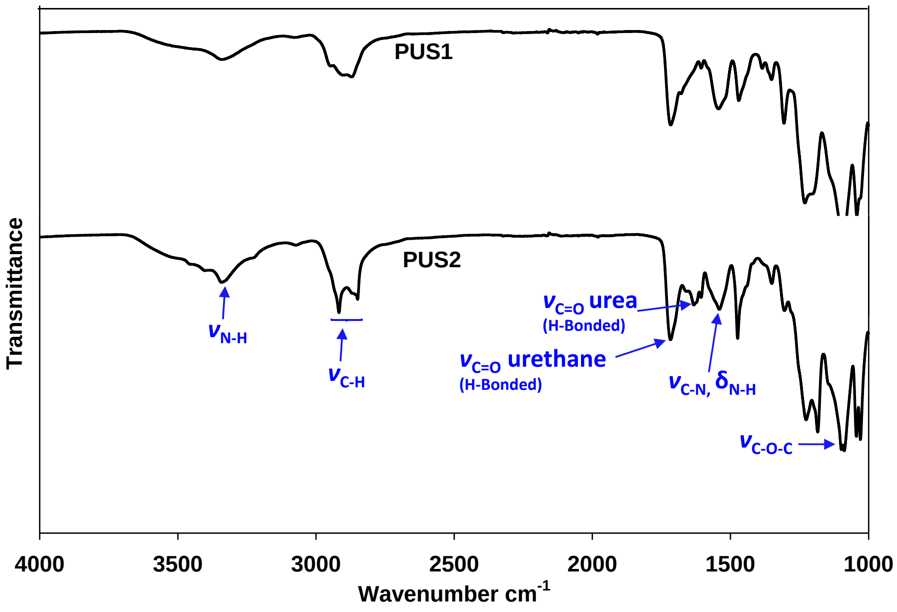

Figure S2: FTIR transmittance spectra of sulfonated polyurethanes (ν = stretching, δ = bending).

Figure S2 shows the characteristic FTIR transmittance plots of the synthesized PUS in the wavelength range of 1000-4000 cm^{-1}. A band around 1540 cm^{-1} corresponding to urethane N-H bending and C-N stretching confirmed the formation of urethane linkage in both polyurethanes. In agreement with literature, hydrogen bonded C=O stretching was observed at 1716 cm^{-1} for PUS1 and at 1717 cm^{-1} for PUS2. A peak corresponding to hydrogen bonded urea C=O stretching at around 1632 cm^{-1} was also present. This peak was more prominent in PUS2 than in PUS1. Furthermore, the absence of a peak at 2250-2270 cm^{-1} corresponding to free isocyanate confirmed the complete consumption of starting materials in both polyurethanes. Furthermore, the two bands observed at 2917 and 2850 cm^{-1} for PUS2 (broad band at ~2872 cm^{-1} for PUS1) were attributed to asymmetric and symmetric stretching, respectively, of the C-H bond of the PEG soft segments. The bands around 1088-1100 cm^{-1} were attributed to C-O-C stretching of ether from PEG and CO-O-C stretching from urethane.

Cytotoxicity Data

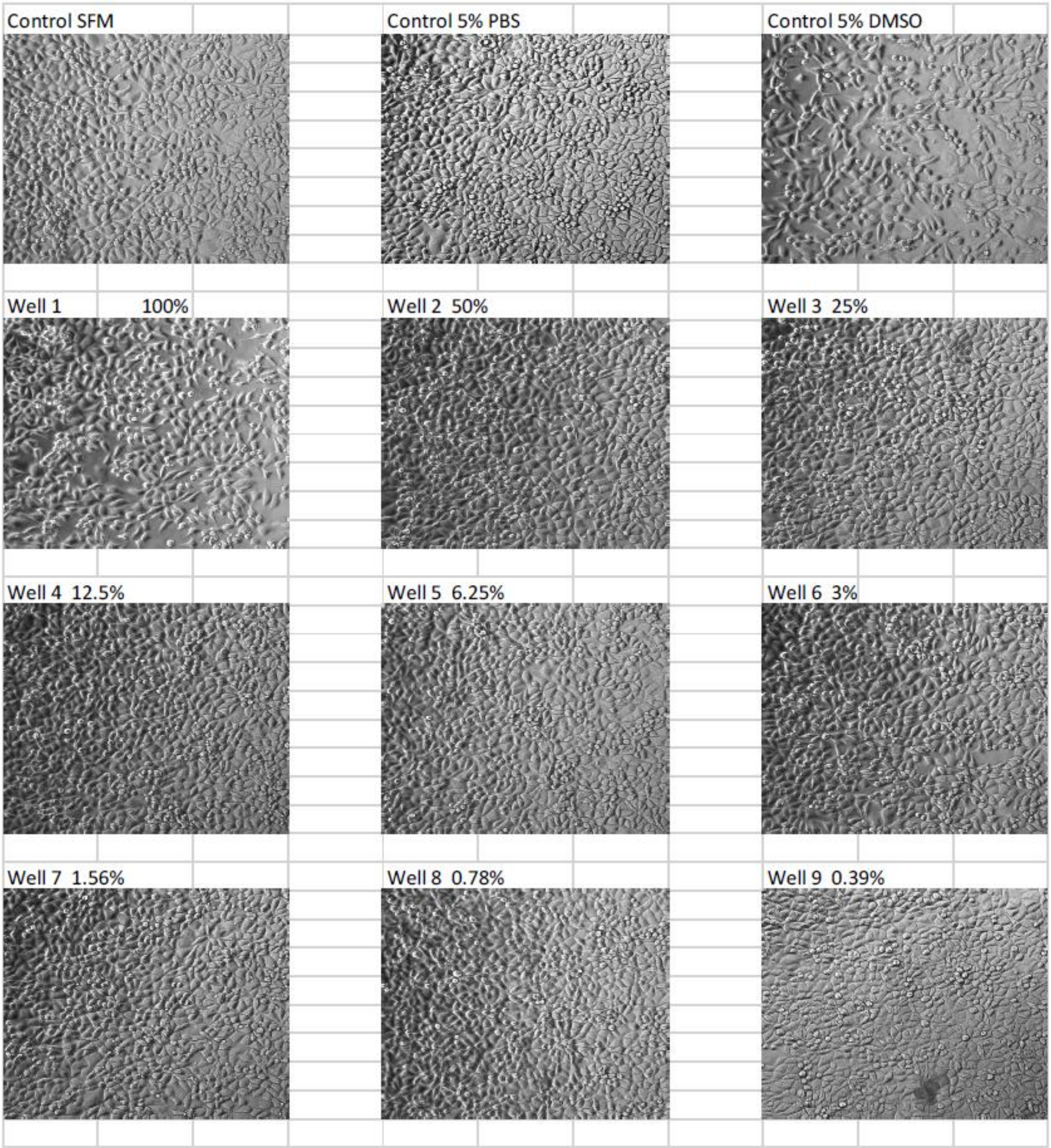

Figure S3: Representative images showing cell morphology after exposure to a diluting series of the PUS2 extract
